# Beyond the Burn: Leukemia Threats Following Radioactive Iodine Ablation Therapy for Thyroid Cancer

**DOI:** 10.3390/cancers17010025

**Published:** 2024-12-25

**Authors:** Mohammad H. Hussein, Eman Toraih, Jessan A. Jishu, Tessa Lavorgna, Ahmed Abdelmaksoud, Ryan Craig, Emad Kandil

**Affiliations:** 1 Division of Endocrine and Oncologic Surgery, Department of Surgery, School of Medicine, Tulane University, New Orleans, LA 70112, USA; 2 Ochsner Clinic Foundation, New Orleans, LA 70112, USA; 3 School of Medicine, Tulane University, New Orleans, LA 70112, USA; 4 Department of Internal Medicine, University of California, Riverside, CA 92521, USA; 5 Department of Pathology and Laboratory Medicine, School of Medicine, Tulane University, New Orleans, LA 70112, USA

**Keywords:** thyroid cancer, leukemia, hematological, radioactive iodine, ablation, RAI, SEER

## Abstract

**Background:** Radioactive iodine (RAI) ablation therapy is a common minimally invasive treatment for patients diagnosed with differentiated thyroid cancer (DTC). Although previous studies have identified a link between RAI and the mortality from secondary solid cancers, the connection between RAI and leukemia remains under-researched. This study investigated the differential risk of leukemia and its subtypes in DTC patients following RAI treatment. **Methods:** DTC patients from the Surveillance, Epidemiology, and End Results (SEER) Registry 17 (2000–2019) were analyzed. The standard incidence ratio (SIR) and excess risk (ER) compared to the reference population were calculated. **Results:** Out of 196,569 DTC patients, 1381 patients developed various types of hematological malignancies. Leukemia was diagnosed in 508 of these patients, and it had the highest risk among the malignancies studied, with an SIR of 1.74 (95%CI: 1.59–1.9). The RAI group had an SIR of 2.12 (95%CI: 1.87–2.39), while the non-RAI group had an SIR was 1.45 (95%CI: 1.37–1.52) (*p* < 0.001). Those diagnosed before the age of 55 years had a conspicuously elevated risk (SIR 2.74) compared to those diagnosed at 55 years or older (SIR 1.53). American Indian/Alaska Native survivors manifested a pronounced leukemia risk with an SIR of 7.63 (95%CI: 2.46–17.8). **Conclusions:** RAI treatment increased the risk of developing leukemia when serving as adjuvant therapy in surgical patients (SIR 2.12). There exists a significant association between RAI treatment in DTC patients and the incidence of leukemia. This susceptibility seems to be modulated by factors including time since diagnosis, age, gender, and racial background.

## Simple Summary

Radioactive iodine ablation therapy is a common treatment for patients with differentiated thyroid cancer. However, recent studies have found that this therapy carries an increased risk for developing leukemia. We compared the risk of different subtypes of leukemia that radioactive iodine ablation therapy may influence as well as other factors that may contribute to such additional risk. Understanding such risks can help guide clinicians in deciding whether to prescribe radioactive iodine therapy to treat their patients and formulating personalized treatment plans.

## 1. Introduction

Differentiated thyroid cancer (DTC) is the most common endocrine cancer in the United States, with its incidence gradually increasing over the past few decades, although it has slightly declined since 2014 [1,2,3,4]. Incidence-based mortality rates have concomitantly increased over recent years, implying the presence of some other advanced disease or cancer not reflected by these declining DTC incidence rates [1].

With thyroidectomy being a primary surgical intervention for patients with DTC, radioactive iodine (RAI) ablation therapy is often used as a remnant therapy to destroy any thyroid tissue that persists after surgery [5,6,7]. It can also be used as an adjuvant therapy to eliminate microscopic cancer cells that may persist after surgery and prevent recurrence [8]. The notion of cells in DTC being able to perform iodine uptake and iodination is the cornerstone of RAI therapy, which has become very popular.

Recent studies have shown that RAI outcomes have been slightly controversial but favorable in post-surgical DTC patients after accounting for risk level and stratification, but the rising incidence-based mortality rates remain a question [9]. With RAI recently being approved by the Food and Drug Administration and considering the increase in such mortality rates, there is a question as to whether an association exists between the two. Pasqual et al. recently studied the link between RAI for DTC in pediatric and young adult populations and the risk of second primary malignancies and found that the cancers with the highest risk (14%) were hematological [10]. Boucai et al. added to such findings in a recent single-institution study that associated RAI with a higher prevalence of clonal hematopoiesis, a precursor for hematological malignancies [11]. There exist several studies that support the relationship between RAI and increased mortality from second primary solid cancers [12,13,14], but, to the best of our knowledge, no studies have been performed that confirm and explain the association between RAI and leukemia as one of these hematological malignancies. As such, the present study sought to examine whether there is a differential risk of developing leukemia and its subtypes following RAI treatment in DTC patients.

## 2. Materials and Methods

### 2.1. Data Source

The Surveillance, Epidemiology, and End Results (SEER) program (https://seer.cancer.gov/), established by the National Cancer Institute (NCI) in 1973, serves as a comprehensive source of cancer statistics in the United States. The incidence data for this study were drawn from the SEER Research Plus Data, 17 Registries (excluding Alaska), November 2021 Submission (covering the years 2000–2019).

### 2.2. Selection of Thyroid Cancer Population

The study cohort was drawn from a population diagnosed with first primary DTC exhibiting malignant behavior, as coded by ICD-O-3/WHO 2008 (N = 203,799 cases). We excluded cases documented only by death certificate (N = 632) and those determined only by autopsy (N = 417). Participants with positive histology confirmation were included (N = 196,569 cases). The study timeframe ranged from January 2000 to December 2019, incorporating a two-month exclusion period post diagnosis. Time-sensitive variables were segregated at intervals for latency (6 months, 1 year, 3 years, 5 years, and 10 years), attained age groups (under 55 and 55 or older), and achieved calendar years (2000, 2005, 2010, 2015, and 2020). The analysis considered a single outcome analysis, with exit points set at events noted in the rate file.

### 2.3. Study Variables

The variables under examination consisted of demographics, pathology TNM, therapy (surgery, radiation, systemic, and RAI), and stratification by calendar year, age group, sex, race, latency course, and radiation therapy.

### 2.4. Primary Outcomes

The primary outcomes involved the calculation of the standard incidence ratio (SIR) and excess risk (ER). The SIR is a crucial tool in the field of cancer research, as it allows for the comparison of hematological cancer incidence rates in DTC patients who underwent RAI to that of a reference population. This ratio is calculated by dividing the observed cases (O) by the expected cases (E) in the study population and multiplying the result by 100 (SIR = (O/E) × 100). Observed cases refer to the number of new cancer cases identified in the study population during a specific period, typically sourced from cancer registries. Expected cases represent the estimated number of new cancer cases in the study population based on the incidence rate of a reference population. This estimate is calculated by applying age-specific incidence rates from the reference population to the age distribution of the study population. Lastly, the reference population serves as the benchmark for comparison and should be representative of the larger population to which the study population belongs while also providing stable and reliable cancer incidence data.

The ER assesses the additional risk of DTC patients with a certain risk factor for developing leukemia after RAI. This assessment determines how influential a specific risk factor may be in leukemia incidence and suggests which risk factors are more distinguished and require special attention. The ER is computed by subtracting the expected cases (E) from the observed cases (O) and dividing this difference by the total population at risk. An ER greater than 0 indicates an increased risk in the study population with the specific risk factor compared to the reference population, while an ER less than 0 indicates a decreased risk. An ER of 0 suggests no additional risk associated with the specific risk factor present in the study population.

### 2.5. Subgroup Analysis

Stratification analysis was performed to investigate whether certain patient characteristics (e.g., age, sex, race/ethnicity, or cancer histology) modify the association between RAI therapy for DTC and hematological cancer risk.

### 2.6. Statistical Analysis

The analysis incorporated the calculation of observed cases (O), expected cases (E), age distribution adjustments, and the use of a reference population to generate the SIR and ER. Additionally, the confidence intervals were established at a 95% level using the approximate method to provide an estimate of the precision of the SIR. SIR and ER values were compared using *p*-values. A *p*-value of <0.05 was considered statistically significant.

## 3. Results

### 3.1. Incidence Rate of Leukemia Following Thyroid Cancer

Out of the 196,569 patients diagnosed with DTC, 1381 patients developed various hematological malignancies. This patient group showed an elevated risk when compared to the general population, as demonstrated by a standardized incidence ratio (SIR) of 1.45 (95%CI: 1.37–1.52). These malignancies encompassed lymphomas, myelomas, and leukemias. Leukemia was found in 508 patients and was observed to have the highest risk among the hematological malignancies studied, with an SIR of 1.74 (95%CI: 1.59–1.9). Aside from Hodgkin lymphoma, DTC patients are at an increased risk for various types of hematopoietic diseases, as elaborated in Supplementary Table S1. The non-lymphocytic leukemia group demonstrated the highest risk among the leukemia subtypes, with the chronic myeloid/monocytic leukemia subtype exhibiting the highest SIR at 2.71 (95%CI = 2.22–3.27) (Table 1).

### 3.2. Dynamic Temporal Trends of Leukemia Following Thyroid Cancer

**Figure 1:**
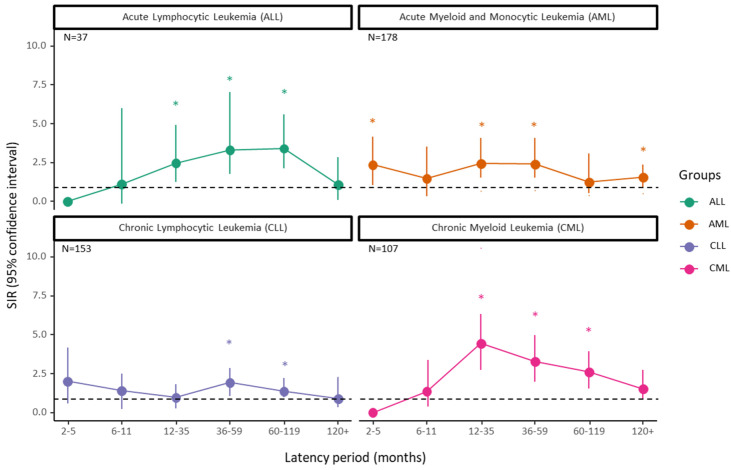
Latency course of developing leukemia following thyroid cancer. Data are stratified according to selected subtypes. SIR: standardized incidence ratio; ALL: acute lymphocytic leukemia; CLL: chronic lymphocytic leukemia; AML: acute myeloid/monocytic leukemia; CML: chronic myeloid leukemia. Points are the estimates of SIR, and error bars represent the 95% confidence interval. The horizontal dotted line stands for SIR at 1.0, indicating similar risk to the general population. Statistically significant values (*p* < 0.05) are denoted by an asterisk sign (*).

The trends of leukemia following DTC show significant changes in the SIR over time, with distinct peaks and troughs for acute lymphocytic leukemia (ALL), acute myeloid/monocytic leukemia (AML), chronic lymphocytic leukemia (CLL), and chronic myeloid/monocytic leukemia (CML) at various latency periods (Figure 1).

### 3.3. Demographic Risk Factors Associated with Leukemia Following Thyroid Cancer

**Figure 2:**
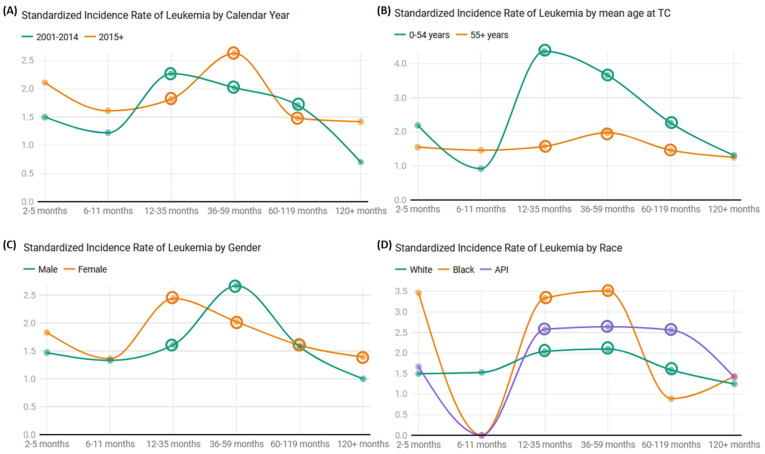
Comparative analysis across time, age, gender, and race. Standardized incidence ratios (SIR) at y-axis and timescales on x-axis across varying demographics. Based on 95% confidence interval, point estimate is circled if the lower and upper limits exceed 1.0.

Our temporal data showed a sustained rise in the SIR values during two distinct phases: 2001–2014 (SIR: 1.79, 95% CI: 1.57–2.02) and 2015 onwards (SIR: 1.7, 95% CI: 1.5–1.93) (Figure 2A).

Age emerged as a formidable determinant, showcasing a striking difference in SIRs between younger patients (0–54 years; SIR: 2.78, 95% CI: 2.33–3.28) and their older counterparts (55+ years; SIR: 1.53, 95% CI: 1.38–1.7). Tracing temporal dynamics displayed significant amplifications in SIR for leukemia among patients aged 0–54 across latency periods, peaking at 12–35 months (SIR: 4.36, 95%CI: 3.23–5.74) and declining to 3.66 (95%CI: 2.53–5.11) and 2.26 (95%CI: 1.57–3.16) at 36–59 months and 60–119 months, respectively.

Similarly, older individuals (55+) revealed augmented SIRs, albeit less intense, across comparable latency intervals: 12–35 months (SIR: 1.57, 95%CI: 1.24–1.97), 36–59 months (SIR: 1.97, 95%CI: 1.57–2.44), and 60–119 months (SIR: 1.46, 95%CI: 1.21–1.75) (Figure 2B).

Parsing the data across genders revealed a balanced risk, with females (SIR: 1.79, 95% CI: 1.6–2) presenting slightly higher SIRs than males (SIR: 1.66, 95% CI: 1.43–1.92). Post thyroid cancer, an escalation in SIRs was discernible across all latency periods in both sexes (Figure 2C).

Our study also cast light on the racial disparities interwoven in the risk profile. Both Black (SIR: 2.04, 95% CI: 1.36–2.93) and Asian or Pacific Islander (SIR: 2.17, 95% CI: 1.48–3.06) populations manifested considerably higher SIRs relative to their White counterparts (SIR: 1.7, 95% CI: 1.54–1.86). Across the racial divide, individuals from all races demonstrated an amplified risk of leukemia after the 12-month mark (Figure 2D).

### 3.4. Leukemia Risk Following Thyroid Cancer Treatment

Table 2 assesses the interplay of demographic factors, pathological risk factors, treatment methods, and subsequent risks of different subtypes of leukemia in DTC survivors. A temporal assessment indicates a modest decrease in leukemia risk among survivors diagnosed from 2015 onward (SIR 1.70, CI: 1.50–1.93) as compared to those diagnosed between 2001 and 2014 (SIR 1.79, CI: 1.57–2.02). This trend is most prominent in the risk dynamics of CML, with a decrease from 3.03 to 2.41.

Those diagnosed before the age of 55 years presented a conspicuously elevated risk (SIR 2.74) compared to those diagnosed at 55 years or older (SIR 1.53). Sexual disparities are also observable, with females slightly edging out their male counterparts. For females, the overall leukemia SIR is 1.79 (CI: 1.60–2.00). However, males displayed an alarmingly higher risk for ALL at an SIR of 3.58 (CI: 2.00–5.90).

Racial disparities in leukemia risk also presented some remarkable findings, with American Indian/Alaska Native survivors manifesting a pronounced leukemia risk with an SIR of 7.63 (CI: 2.46–17.8). Regarding ethnicity, non-Spanish-Hispanic-Latino survivors have the highest risk, registering an SIR of 1.75 (CI: 1.59–1.92).

Among the histological variants of DTC, PTC and FTC survivors showed comparably elevated risks, having SIRs of 1.90 (CI: 1.67–2.16) and 1.96 (CI: 1.28–2.87), respectively.

From a therapeutic perspective, those who did not undergo cancer-directed surgery had an SIR of 1.74 (CI: 1.59–1.90) compared to 1.34 (CI: 0.58–2.64) for those who did. RAI was particularly revealing in survivors who abstained, as they exhibited an SIR of 1.45 (CI: 1.26–1.65), significantly lower than those who underwent the procedure with an SIR of 2.12 (CI: 1.87–2.39).

### 3.5. Stratified Analysis for Risk of Leukemia Following Radioactive Ablation Iodine Therapy

Table 3 examines the risk of leukemia in cohorts of patients diagnosed with DTC who received RAI and those who did not. For the overall cohort, the observed number of leukemia cases among the non-RAI group was 225, with an SIR of 1.45 (95% CI: 1.26–1.65) and an ER of 0.98. In contrast, the RAI group had 260 cases, with a notably higher SIR of 2.12 (95% CI: 1.87–2.39) and an ER of 2.12 (*p* < 0.001).

**Figure 3:**
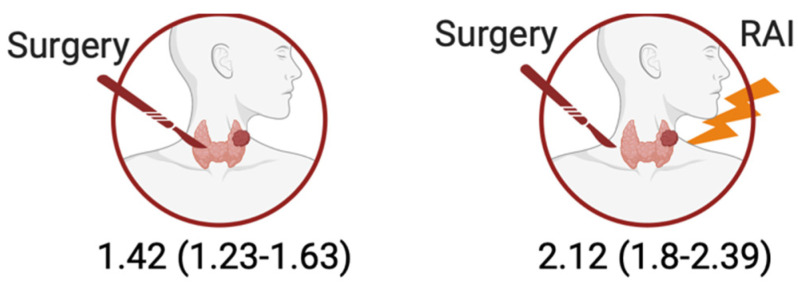
Standardized incidence ratios of leukemia after RAI in surgical patients.

Out of 467 cases that underwent thyroid surgery, 259 cases received RAI therapy as an adjuvant therapy after surgery. Such therapy was found to have an increased risk of developing leukemia. This risk was lowered in surgical patients who did not receive any RAI treatment (Figure 3).

## 4. Discussion

The increasing rates of both the incidence of DTC and use of RAI for it over the past few decades make it important to study the outcomes of this treatment. This importance is augmented by recent studies that have found both beneficial and harmful results of RAI, including a heightened risk of leukemia. The present study is one of the very few to comprehensively explain this association in the United States and, to the best of our knowledge, the very first to identify associations with both lymphocytic and myeloid leukemias. Overall, we found that DTC patients who underwent RAI had a greater risk of developing leukemia (SIR 2.12) compared to those who did not (SIR 1.45), with RAI being attributed to a significantly greater risk of developing leukemia (ER 2.12).

Overall, our findings corroborate the influence RAI treatment has on the development of leukemia [15,16,17]. However, they expand upon this general association by examining the subtypes of leukemia, revealing that there are significantly heightened risks of developing both lymphocytic and myeloid leukemias. The former association is surprising given that lymphocytic leukemia is generally regarded to not be caused by ionizing radiation and that the risk of developing this specific subtype was not associated with RAI treatment [10]. However, other studies have argued against the non-radiogenic nature of lymphocytic leukemia given the poor epidemiological data and have even suggested the possibility that it may indeed be radiogenic [18,19]. Nevertheless, this study is the first to identify associations between RAI and all major subtypes of leukemia.

RAI can be administered as remnant ablation, adjuvant therapy, or treatment of persistent disease, with the 2015 American Thyroid Association guidelines generally recommending adjuvant therapy after total thyroidectomy for high-risk patients and not for low-risk patients [20]. Although these guidelines strictly regulate when RAI is indicated, recent analyses of institutional databases have found that 20–44% of low-risk cases of DTC eligible for lobectomy may eventually need completion thyroidectomy due to pathologic findings that suggest the need for RAI under the current guidelines [21,22]. Although our study confirmed that the risk of developing leukemia increases when RAI is used as adjuvant therapy, there is limited evidence in the literature that compares this risk after receiving single surgery (total thyroidectomy) to staged surgery (lobectomy leading to completion thyroidectomy). As such, associations between RAI following each type of thyroid surgery and the risk of leukemia should be examined in future studies.

We also confirmed that younger age is more associated with developing leukemia following RAI [23,24]. Interestingly, we found a consistent and statistically significant increase in the SIR for leukemia for every five-year age increment until the age of 55, which was deemed an appropriate age cutoff. This holds important implications in the quality of life of children, adolescents, and young adults, especially since DTC is quite common in that population. These findings demonstrate the heightened vulnerability of younger individuals to leukemia in the aftermath of DTC and should advise physicians to strongly consider the possible repercussions of RAI in their pediatric and young adult DTC patients.

Additionally, leukemia incidence appeared to be higher in minority races, specifically in the Black and API populations compared to the White population. Shah et al. revealed that Black patients historically underwent more appropriate RAI therapy and had a higher probability of being under-treated with RAI than their White counterparts, even though over-treatment increased slightly over time [25]. Therefore, the frequency and intensity of RAI therapy were not likely to influence this finding. Furthermore, there is generally a lower incidence of DTC in the Black population [26], implying that the higher leukemia incidence in this population may be clinically important, underscoring a compelling need for targeted risk-mitigation strategies in this vulnerable population.

Unfortunately, the SEER database lacks information regarding the doses of RAI used, precluding us from determining any formal association between RAI dose and risk of developing leukemia. Indeed, the dose required is generally dependent on the intensity of the cancer and requires thorough, patient-centered decision making. Hong et al. summarized the factors that influence the decision making of which empirical RAI dose to use, which include the pathologic factors of the cancer itself as well as miscellaneous factors such as surgeon experience, type of surgical procedure, and individual patient characteristics [27]. Such factors should be considered given the previous studies that have suggested the strong influence that dose has on the incidence of leukemia. Rubino et al. studied the risks of second primary malignancies in DTC patients and found that the excess absolute risk was 0.8 cases of leukemias per 1 GBq (or approximately 27 mCi) of RAI used in 10,000 person-years [28]. More recently, Seo et al. and Hailan et al. found in their large population-based and systematic reviews that RAI od doses exceeding 100 mCi was associated with the incidence of leukemia, while that of lower doses was not [29,30]. Despite these findings, there is still no RAI dose threshold defined in the literature. Reasons for this may include the limited studies available as well as the different roles RAI has, such as remnant therapy or adjuvant therapy. Although we confirmed the association between RAI therapy and risk of developing leukemia, further studies are required to investigate the influence of the dose.

Although the present study demonstrates great clinical relevance, it comes with limitations. The SIR is a key statistic generated by the SEER program and provides insights into the incidence of specific cancers in the population. The interpretation of the SIR should be performed with caution, as it may be influenced by various factors, such as differences in data quality, completeness of case ascertainment, and changes in diagnostic practices. Additionally, we were unable to assess the correlation between RAI treatment and the development of leukemia based on the molecular or genetic basis of DTC. We recommend further studies to investigate if the risk of developing leukemia after RAI treatment is heightened in the presence of any specific mutations, such as *BRAF* or *KRAS*. Moreover, statistical fluctuations may also affect the SIR, particularly for rare cancers or small populations. Future studies can add to these findings by explaining how DTC patients’ socioeconomic statuses can influence their decisions to undergo RAI and their risks of developing leukemia as a result.

## 5. Conclusions

This is the first study to prove significant associations between RAI treatment in DTC patients and the occurrence of all major individual subtypes of lymphocytic and myeloid leukemia. With DTC becoming more diagnosed and RAI being more utilized as a treatment over the years, the impact on clinical practice is evident and forces physicians to consider leukemia as a very possible consequence of treatment. These findings illuminate the intricate tapestry of leukemia risk in DTC survivors, drawing attention to the multifaceted interplay of time, age, sex, and race. They also motivate the need to consider the economic implications for patients and hospitals that come with treating leukemia. All in all, these insights signal the need for a tailored, demographic-sensitive approach in managing the therapeutic trajectory of DTC patients, thereby enhancing their long-term health outcomes.

## Figures and Tables

**Table 1:** Incidence rate of diverse types of leukemia among the thyroid cancer cohort.

Leukemia Subtype	Count	Percentage	SIR (O/E)	LL	UL	ER
Lymphocytic Leukemia (N = 198)						
Acute Lymphocytic Leukemia	37	18.69	2.46 #	1.73	3.39	0.16
Chronic Lymphocytic Leukemia	153	77.27	1.31 #	1.11	1.54	0.26
Other Lymphocytic Leukemia	8	4.04	1.0	0.43	1.97	0.0
Myeloid Leukemia (N = 285)						
Acute Myeloid/Monocytic Leukemia	178	62.46	1.80 #	1.55	2.09	0.56
Chronic Myeloid/Monocytic Leukemia	107	37.54	2.71 #	2.22	3.27	0.48
Other Myeloid/Monocytic Leukemia	5	1.75	1.69	0.55	3.95	0.01
Other Leukemia (N = 25)						
Other Acute Leukemia	5	20.0	1.20	0.39	2.8	0.01
Aleukemic, Subleukemic and NOS	20	80.0	1.81 #	1.11	2.8	0.06

SIR: standardized incidence ratio; O/E: observed/expected; LL: lower limit of 95% confidence interval; UL: upper limit of 95% confidence interval; ER: excess risk per 10,000. Statistically significant values (*p* < 0.05) are denoted by a pound sign (#).

**Table 2:** Incidence rate of various types of leukemia among the thyroid cancer cohort.

Risk Factor	Leukemia	ALL	CLL	AML	CML
**Demographic data**					
Calendar year					
2001–2014	**1.79 (1.57–2.02)**	**2.02 (1.13–3.33)**	1.21 (0.94–1.52)	**2.08 (1.69–2.53)**	**3.03 (2.30–3.92)**
2015+	**1.70 (1.50–1.93)**	**2.90 (1.82–4.40)**	**1.42 (1.13–1.77)**	**1.54 (1.22–1.93)**	**2.41 (1.78–3.19)**
Age at diagnosis of TC					
<55 years	**2.74 (2.29–3.24)**	**2.06 (1.06–3.60)**	1.62 (0.96–2.56)	**3.07 (2.32–4.00)**	**4.53 (3.28–6.10)**
≥55 years	**1.53 (1.38–1.70)**	**2.67 (1.71–3.97)**	**1.28 (1.07–1.52)**	**1.51 (1.25–1.80)**	**2.14 (1.65–2.73)**
Sex					
Male	**1.66 (1.43–1.92)**	**3.58 (2.00–5.90)**	**1.31 (1.01–1.67)**	**1.54 (1.15–2.02)**	**2.22 (1.52–3.14)**
Female	**1.79 (1.60–2.00)**	**2.03 (1.27–3.08)**	**1.32 (1.06–1.62)**	**1.94 (1.62–2.31)**	**2.99 (2.35–3.75)**
Race					
White	**1.70 (1.54–1.86)**	**2.00 (1.31–2.94)**	**1.29 (1.08–1.52)**	**1.85 (1.57–2.16)**	**2.53 (2.03–3.12)**
Black	**2.04 (1.36–2.93)**	3.03 (0.34–10.9)	1.68 (0.72–3.31)	1.92 (0.92–3.53)	**3.80 (1.73–7.21)**
AI/AN	**7.63 (2.46–17.8)**	**33.9 (3.8–122.4)**	9.10 (0.12–50.6)	2.99 (0.04–16.6)	9.44 (0.12–52.5)
Asian or Pacific Islander	**2.17 (1.48–3.06)**	**5.87 (2.35–12.1)**	1.54 (0.41–3.94)	1.32 (0.63–2.42)	**4.31 (2.06–7.92)**
Ethnicity					
Non-Spanish-Hispanic-Latino	**1.75 (1.59–1.92)**	**2.18 (1.45–3.15)**	**1.41 (1.19–1.66)**	**1.78 (1.51–2.09)**	**2.63 (2.12–3.23)**
Spanish-Hispanic-Latino	**1.67 (1.27–2.15)**	**4.15 (1.89–7.88)**	0.58 (0.25–1.14)	**1.95 (1.23–2.92)**	**3.23 (1.85–5.25)**
**Pathological features**					
Histological variant					
PTC	**1.90 (1.67–2.16)**	**2.59 (1.53–4.09)**	**1.61 (1.27–2.01)**	**1.75 (1.37–2.19)**	**2.76 (2.04–3.66)**
FTC	**1.96 (1.28–2.87)**	1.94 (0.84–3.83)	0.55 (0.11–1.62)	**3.57 (2.04–5.79)**	2.27 (0.61–5.82)
MTC	1.56 (0.67–3.08)	8.97 (1.01–32.3)	0.93 (0.10–3.34)	1.19 (0.13–4.29)	3.01 (0.34–10.9)
T stage					
T1 stage	**1.53 (1.30–1.79)**	1.77 (0.85–3.26)	1.29 (0.96–1.69)	**1.82 (1.40–2.33)**	**1.84 (1.20–2.69)**
T2 stage	**1.97 (1.50–2.55)**	2.96 (0.95–6.91)	1.14 (0.61–1.95)	**1.81 (1.07–2.86)**	**3.85 (2.20–6.26)**
T3 stage	**2.15 (1.70–2.69)**	2.64 (0.85–6.16)	1.42 (0.86–2.19)	**2.32 (1.54–3.35)**	**3.86 (2.32–6.03)**
T4a stage	1.51 (0.65–2.97)	4.26 (0.06–23.7)	0.00 (0.00–1.70)	2.22 (0.60–5.68)	2.88 (0.32–10.4)
T4b stage	0.72 (0.08–2.59)	0.00 (0.00–31.6)	0.00 (0.00–3.18)	1.06 (0.01–5.88)	2.77 (0.04–15.4)
N stage					
N0 stage	**1.56 (1.37–1.78)**	**1.96 (1.09–3.23)**	**1.21 (0.94–1.52)**	**1.68 (1.34–2.09)**	**2.35 (1.73–3.13)**
N1a stage	**2.29 (1.61–3.16)**	2.82 (0.57–8.23)	1.16 (0.46–2.39)	**3.48 (2.09–5.43)**	**2.98 (1.19–6.13)**
N1b stage	**2.35 (1.56–3.39)**	4.20 (0.84–12.2)	1.74 (0.75–3.43)	1.51 (0.55–3.28)	**5.32 (2.43–10.1)**
M stage					
M0 stage	**1.72 (1.53–1.93)**	**2.18 (1.35–3.33)**	**1.25 (1.00–1.54)**	**1.93 (1.59–2.31)**	**2.58 (1.98–3.30)**
M1 stage	**2.52 (1.01–5.19)**	0.00 (0.0–32.34)	1.77 (0.20–6.38)	3.14 (0.63–9.17)	5.55 (0.62–20.0)
**Treatment**					
Cancer-directed surgery					
Negative	**1.74 (1.59–1.90)**	**2.47 (1.73–3.41)**	**1.29 (1.09–1.52)**	**1.80 (1.54–2.09)**	**2.75 (2.25–3.33)**
Positive	1.34 (0.58–2.64)	4.21 (0.06–23.4)	0.82 (0.09–2.96)	0.98 (0.11–3.55)	2.57 (0.29–9.29)
Radioactive ablation					
Negative	**1.45 (1.26–1.65)**	1.30 (0.62–2.40)	1.24 (0.98–1.55)	**1.56 (1.24–1.93)**	**1.82 (1.29–2.50)**
Positive	**2.12 (1.87–2.39)**	**3.71 (2.40–5.48)**	**1.41 (1.09–1.79)**	**2.16 (1.74–2.66)**	**3.74 (2.87–4.78)**
Systemic therapy					
Negative	**1.80 (1.54–2.09)**	1.86 (0.85–3.54)	**1.56 (1.19–2.01)**	**1.71 (1.30–2.23)**	**2.61 (1.81–3.64)**
Positive	**1.82 (1.63–2.26)**	**2.82 (1.45–4.92)**	1.33 (0.95–1.82)	**2.06 (1.54–2.66)**	**3.28 (2.29–4.56)**

Data are reported as standardized incidence ratios (SIR) with 95% confidence intervals. ALL: acute lymphocytic leukemia; CLL: chronic lymphocytic leukemia; AML: acute myeloid/monocytic leukemia; CML: chronic myeloid leukemia; AI/AN: American Indian/Alaska Native. Statistically significant values (*p* < 0.05) are denoted by bold values.

**Table 3:** Comparative analysis of leukemia risk following radioactive iodine treatment in thyroid cancer patients.

Risk Factor	No RAI			RAI			*p*-Value
	Observed	SIR (95%CI)	ER	Observed	SIR (95%CI)	ER	
**Overall analysis**							
All cohorts	225	1.45 (1.26–1.65)	0.98	260	**2.12 (1.8–2.39)**	2.12	**<0.001**
**Demographic data**							
Age at diagnosis of TC							
<55 years	48	**2.05 (1.51–2.72)**	0.7	81	**3.42 (2.7–4.25)**	1.56	**0.015**
≥55 years	177	**1.34 (1.15–1.55)**	1.26	179	**1.81 (1.5–2.09)**	2.86	**0.006**
Sex							
Male	73	**1.33 (1.04–1.67)**	1.24	101	**2.06 (1.68–2.51)**	3.38	**0.011**
Female	152	**1.51 (1.28–1.77)**	0.91	159	**2.16 (1.8–2.52)**	1.73	**0.003**
Race							
White	199	**1.44 (1.24–1.65)**	1.04	223	**2.04 (1.8–2.32)**	2.14	0.57
Black	15	**1.78 (1.00–2.94)**	1.34	12	**2.34 (1.2–4.08)**	1.99	0.51
AI/AN	8	1.19 (0.51–2.34)	0.19	22	**3.00 (1.8–4.55)**	1.96	0.13
Asian or Pacific Islander	3	**8.47 (1.70–24.74)**	7.6	2	7.71 (0.8–27.8)	4.77	0.94
**Pathological features**							
Histological variant							
PTC	99	**1.56 (1.27–1.90)**	1.13	128	**2.25 (1.8–2.68)**	2.17	0.012
FTC	8	1.33 (0.57–2.61)	0.78	17	**2.64 (1.5–4.23)**	3.88	0.233
MTC	5	1.11 (0.36–2.58)	0.3	1	9.19 (0.1–51.1)	21.93	**0.004**
T stage							
T1 stage	114	**1.35 (1.12–1.62)**	0.79	83	**1.99 (1.58–2.4)**	1.82	**0.008**
T2 stage	29	**2.07 (1.39–2.98)**	2.25	43	**2.10 (1.5–2.83)**	2.01	0.95
T3 stage	19	1.36 (0.82–2.13)	0.87	70	**2.47 (1.9–3.12)**	3.09	0.07
T4a stage	3	1.96 (0.39–5.73)	2.81	7	1.85 (0.7–3.82)	2.14	0.94
T4b stage	1	1.2 (0.02–6.68)	0.63	3	1.72 (0.3–5.04)	1.95	0.85
N stage							
N0 stage	146	**1.37 (1.16–1.61)**	0.86	141	**2.00 (1.6–2.36)**	2.07	**0.002**
N1a stage	9	1.72 (0.78–3.26)	1.25	33	**2.42 (1.6–3.39)**	2.24	0.45
N1b stage	7	1.75 (0.7–3.6)	1.67	25	**2.59 (1.6–3.82)**	2.76	0.42
M stage							
M0 stage	171	**1.46 (1.25–1.69)**	1.03	204	**2.13 (1.8–2.45)**	2.18	**0.019**
M1 stage	0	0 (0–3.9)	−3.34	6	**3.38 (1.2–7.36)**	7.11	NA
**Treatment**							
Cancer-directed surgery							
Negative	8	1.58 (0.68–3.11)	1.61	6	**2.80 (1.02–6.1)**	4.45	0.37
Positive	208	**1.42 (1.23–1.63)**	0.91	259	**2.12 (1.8–2.39)**	2.12	**<0.001**
**Latency**							
2–5 months	12	1.87 (0.97–3.27)	1.65	7	1.67 (0.7–3.45)	1.06	0.83
6–11 months	12	1.31 (0.67–2.28)	0.58	9	1.46 (0.6–2.76)	0.73	0.82
12–35 months	51	**1.54 (1.14–2.02)**	1.06	71	**3.01 (2.3–3.79)**	3.36	**0.002**
36–59 months	49	**1.75 (1.29–2.31)**	1.58	63	**2.93 (2.2–3.74)**	3.46	**0.017**
60–119 months	60	1.23 (0.94–1.58)	0.53	82	**2.00 (1.5–2.49)**	1.99	**0.011**
120+ months	41	1.38 (0.99–1.87)	0.98	28	1.06 (0.7–1.54)	0.15	0.32

Data are reported as standardized incidence ratios (SIR) with 95% confidence intervals and excess risk (ER). AI/AN: American Indian/Alaska Native. Statistically significant values (*p* < 0.05) are denoted by bold values.

## Data Availability

Publicly available datasets were analyzed in this study. These data can be found here: https://seer.cancer.gov.

## Supplementary Materials

The following supporting information can be downloaded at: https://www.mdpi.com/article/10.3390/cancers17010025/s1, Table S1: Incidence rate of various types of lymphatic and hematopoietic diseases among TC cohort.
