# Supplementary material for: Beyond the Burn: Leukemia Threats Following Radioactive Iodine Ablation Therapy for Thyroid Cancer
Source: Cancers (Basel). 2024 Dec 25;17(1):25. doi: 10.3390/cancers17010025 (PMC11718960; doi:10.3390/cancers17010025)
Supplement: Supplementary file 1 [file cancers-17-00025-s001.zip › cancers-3347438-supplementary.pdf]

Supplementary Table S1. Incidence rate of various types of lymphatic and hematopoietic diseases among TC cohort.

Type of Cancer	Count	Percentage	SIR (O/E)	LL	UL	Excess risk
All Lymphatic and Hematopoietic Diseases	1,381	0.70	1.45#	1.37	1.52	3.01
Lymphoma	645	0.33	1.29#	1.19	1.39	1.01
Hodgkin Lymphoma	36	0.02	0.95	0.67	1.32	-0.01
Non-Hodgkin Lymphoma	609	0.31	1.31#	1.21	1.42	1.03
Myeloma	228	0.12	1.41#	1.23	1.6	0.47
Leukemia	508	0.26	1.74#	1.59	1.9	1.53
Lymphocytic Leukemia	198	0.10	1.42#	1.23	1.63	0.41
Myeloid Leukemia	285	0.14	2.08#	1.85	2.34	1.05
Other Leukemia	25	0.01	1.64#	1.06	2.43	0.07

SIR: standardized incidence ratio, O/E: observed/expected. The SIR is a measure used to compare the cancer incidence of a specific population to that of the general population. It is calculated by dividing the observed number of cancer cases in the population of interest by the expected number of cases based on the general population's rates. An SIR greater than 1.0 indicates that the number of observed cases is higher than expected, an SIR less than 1.0 means fewer cases were observed than expected, and an SIR of 1.0 suggests that the number of observed cases is equal to the expected number. The accompanying lower limit (LL) and upper limit (UL) values establish the range within which the true SIR value likely resides, with a 95% certainty. Excess risk is a measure of the additional risk a certain population has of developing a specific disease compared to the general population. In the context of SEER, this is usually given per 10,000 person-years at risk. This means that, for every 10,000 individuals in the population of interest observed over one year, the 'excess risk' number of additional cases would be expected compared to what would be seen in a comparable general population. Statistically significant values ($p < 0.05$) are denoted by a pound sign (#).
